# Machine learning classification of texture features of MRI breast tumor and peri-tumor of combined pre- and early treatment predicts pathologic complete response

**DOI:** 10.1186/s12938-021-00899-z

**Published:** 2021-06-28

**Authors:** Lal Hussain, Pauline Huang, Tony Nguyen, Kashif J. Lone, Amjad Ali, Muhammad Salman Khan, Haifang Li, Doug Young Suh, Tim Q. Duong

**Affiliations:** 1 Department of Computer Science & IT, Neelum Campus, The University of Azad Jammu and Kashmir, Muzaffarabad, Azad Kashmir Pakistan; 2 Department of Computer Science & IT, King Abdullah Campus, The University of Azad Jammu and Kashmir, Muzaffarabad, Azad Kashmir Pakistan; 3 Department of Radiology, Renaissance School of Medicine At Stony, Brook University, 101 Nicolls Rd, Stony Brook, NY 11794 USA; 4 Department of Computer Science, COMSATS University Islamabad, Lahore Campus, Lahore, Pakistan; 5 College of Electronics and Convergence Engineering, Kyung Hee University, Seoul, South Korea; 6 Department of Radiology, Albert Einstein College of Medicine and Montefiore Medical Center, 111 East 210th Street, Bronx, NY 10467 USA

**Keywords:** Molecular subtypes, Neoadjuvant chemotherapy, Magnetic resonance imaging, Artificial intelligence, Texture features, Radiomics

## Abstract

**Purpose:**

This study used machine learning classification of texture features from MRI of breast tumor and peri-tumor at multiple treatment time points in conjunction with molecular subtypes to predict eventual pathological complete response (PCR) to neoadjuvant chemotherapy.

**Materials and method:**

This study employed a subset of patients (*N* = 166) with PCR data from the I-SPY-1 TRIAL (2002–2006). This cohort consisted of patients with stage 2 or 3 breast cancer that underwent anthracycline–cyclophosphamide and taxane treatment. Magnetic resonance imaging (MRI) was acquired pre-neoadjuvant chemotherapy, early, and mid-treatment. Texture features were extracted from post-contrast-enhanced MRI, pre- and post-contrast subtraction images, and with morphological dilation to include peri-tumoral tissue. Molecular subtypes and Ki67 were also included in the prediction model. Performance of classification models used the receiver operating characteristics curve analysis including area under the curve (AUC). Statistical analysis was done using unpaired two-tailed *t*-tests.

**Results:**

Molecular subtypes alone yielded moderate prediction performance of PCR (AUC = 0.82, *p* = 0.07). Pre-, early, and mid-treatment data alone yielded moderate performance (AUC = 0.88, 0.72, and 0.78, *p* = 0.03, 0.13, 0.44, respectively). The combined pre- and early treatment data markedly improved performance (AUC = 0.96, *p* = 0.0003). Addition of molecular subtypes improved performance slightly for individual time points but substantially for the combined pre- and early treatment (AUC = 0.98, *p* = 0.0003). The optimal morphological dilation was 3–5 pixels. Subtraction of post- and pre-contrast MRI further improved performance (AUC = 0.98, *p* = 0.00003). Finally, among the machine-learning algorithms evaluated, the RUSBoosted Tree machine-learning method yielded the highest performance.

**Conclusion:**

AI-classification of texture features from MRI of breast tumor at multiple treatment time points accurately predicts eventual PCR. Longitudinal changes in texture features and peri-tumoral features further improve PCR prediction performance. Accurate assessment of treatment efficacy early on could minimize unnecessary toxic chemotherapy and enable mid-treatment modification for patients to achieve better clinical outcomes.

## Background

Neoadjuvant chemotherapy (NAC) [1] is often given to patients with breast cancer prior to surgical excision of the tumor in order to reduce the tumor size and minimize risk of distant metastasis. For assessing the treatment response at the end of NAC, the pathological complete response (PCR) [2, 3], which is defined as absence of invasive cancer in the axillary lymph nodes and breast, is the standard. Patients who achieve PCR are more likely to be the candidate of breast conserving surgery and have longer overall survival and recurrence-free survival [2, 3]. It is of clinical importance to know early in the NAC process whether the patient will respond, because clinicians can then adjust medications or choose alternative methods such as hormone therapy or radiation therapy and discontinue ineffective chemotherapy. For this reason, it is desirable to predict PCR using pre- and early treatment data instead of waiting months until the end of NAC to know if the treatment was effective. MRI is an attractive non-invasive method of monitoring treatment progress because it provides good soft tissue contrast and a high-resolution 3D view of the whole breast. Texture analysis and machine learning is capable of processing MRI images and extracting patterns that may be indiscernible to the human eye.

Many studies have utilized molecular subtypes [4–6], tumor volume [4, 7], and breast tumor radiomics [8–17] at initial time points to predict eventual PCR. Molecular subtypes of breast cancer play an important role in informing whether patients are more likely to respond to NAC. However, by themselves, they do not have sufficient accuracy to predict eventual PCR [4–6]. Radiological assessment and tumor volume also do not have sufficient accuracy to predict eventual PCR either [7]. Furthermore, a few studies have used texture analysis and machine learning [8–17] of breast MRI to predict PCR. However, most of these texture analysis studies analyzed the contrast-enhanced tumor alone. Peritumoral microenvironment, in addition to the tumor, could play an important role in cancer development and chemoresistance [18]. Machine learning may also be able to detect early subtle changes in peri-tumoral microenvironment which may improve PCR prediction accuracy. To our knowledge, there are no published studies using machine learning analysis of tumoral and peri-tumoral texture features at multiple treatment time points that include molecular markers and patient demographic data to predict PCR.

The goal of this study was to determine whether machine learning classification of texture features of breast MRI, in conjunction with molecular subtypes, could accurately predict PCR associated with NAC in breast cancer. We performed PCR prediction on: (i) texture features at individual treatment time points and combination of time points (manually segmented tumor from post-contrast MRI); (ii) MRI tumor texture features + molecular subtypes; (iii) tumor with peri-tumor using automated graded morphological dilation, and (iv) post- and pre-contrast subtraction MRI with and without “dilation”. We also compared the prediction performance of 5 machine-learning classifiers: Ensemble, *K*-nearest neighbor, support vector machine, Naïve Bayes, and Decision Tree.

## Results

In Table 1, a subset of the I-SPY-1 patient pool with PCR and MRI data is analyzed. For comparison, the molecular subtypes of the entire data of the I-SPY-1 data are shown. The % prevalence of molecular subtypes in this subset was similar to the parent dataset.

**Table 1:** Molecular subtypes of breast cancer for those with PCR and the entire data ISPY-1 data set

Characteristics	PCR dataset *n* = 166 (%)	I-SPY 1 available data *n* = 221 (%)
Age ± SD (years)	48.20 ± 8.88	48.25 ± 8.89
Caucasian		165 (74.66%)
African American		42 (19.00%)
Asian		9 (4.07%)
Native Hawaiian/Pacific Islander		1 (0.45%)
American Indian/Alaskan Native		0 (0.00%)
Multiple race		2 (0.90%)
ER		
ER+	95 (57.23)	125 (56.60)
ER−	71 (42.77)	94 (42.53)
Missing		2 (0.90)
PgR		
PgR+	76 (45.78)	104 (47.05)
PgR−	90 (54.22)	117 (52.94)
Missing		2 (0.90)
HR		
HR+	100 (60.24)	131 (59.28)
HR−	66 (39.76)	90 (40.72)
Missing		2 (0.90)
HER2		
HER2+	49 (29.52)	67 (30.32)
HER2−	117 (70.48)	149 (6.74)
Missing		5 (2.26)
3 level HR/HER2		
HR+/HER2−	74 (44.57)	96 (43.44)
HER2+	49 (29.51)	67 (30.32)
Triple−	40 (24.09)	53 (23.99)
Missing		5 (2.26)

*ER* estrogen receptor, *PgR* progesterone receptor, *HR* hormone receptor, *HER2* human epidermal growth factor receptor 2, *Ki67* a cellular marker for proliferation

**Fig. 1:**
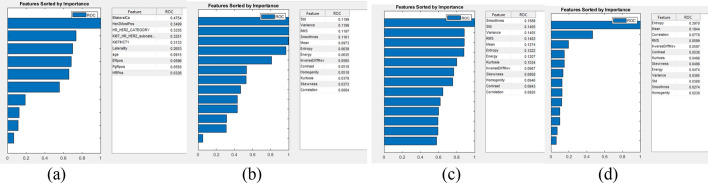
Ranking parameters for **a** molecular subtypes, post-contrast MRI texture at **b** tp1, **c** tp2, and **d** tp1 + tp2

Figure 1 shows the ranking of clinical features, post-contrast MRI texture at tp1, tp2, and tp1 + tp2. The top four clinical features were: the presence of bilateral cancer, HER2 positivity, and HR/HER2+, and Ki67. For tp1, the top four MRI texture features were: standard deviation, variance, root mean square, and smoothness. For tp2, smoothness, standard deviation, variance, and root mean square. For tp1 + tp2, entropy, mean, correlation, and root mean square. Note that the highly ranked features might be correlated, and thus not all highly ranked features would contribute to improving prediction performance.

Table 2 shows the PCR prediction performance analysis on the tumor contour based on the post-contrast MRI with morphological dilation. The AUC of PCR prediction using molecular subtype was 0.82. The AUC of PCR prediction using MRI texture only at tp1, tp2, and tp3 were 0.88, 0.72, and 0.78, respectively. By contrast tp1 + tp2 yielded markedly better performance with an AUC of 0.96. The AUC of MRI texture + molecular subtypes further improved AUC slightly for individual time points and substantially for the combination time points. The *p*-values also became comparatively smaller for the combination time points. We did not perform texture analysis on data for tp4 because the tumor had markedly shrunk or mostly disappeared for most patients. Similar conclusions were reached for most other performance measures (i.e., sensitivity, specificity, etc.). We also performed tp1 + tp3 and tp2 + tp3 (data not shown), and tp1 + tp2 was the best performer among any paired time point combination.

**Table 2:** ROC metrics for predicting PCR based on molecular subtypes, MRI features at pre- and during NAC using Ensemble RUSBoosted Tree classifier

Time point	Features type	Sens.	Spec.	PPV	NPV	Accuracy	AUC	*P*-value
–	Molecular subtypes	86.48	76.92	91.42	66.66	84	0.82 (0.66, 0.97)	0.07
Tp1	MRI texture only	86.48	84.62	94.12	68.75	86	0.88 (0.77, 1.0)	0.03
Tp2		97.30	38.46	81.82	83.33	82	0.72 (0.53, 0.91)	0.13
Tp3		92.85	30	78.78	60	76	0.78 (0.62, 0.95)	0.44
Tp1 + Tp2		1.00	76.92	92.50	1.00	84	0.96 (0.92, 1.0)	0.0003
Tp1	MRI texture + molecular subtypes	89.18	92.30	97.06	75.00	90	0.86 (0.75, 0.98)	0.005
Tp2		89.18	69.23	89.18	69.23	84	0.80 (0.64, 0.96)	0.068
Tp3		96.42	50	84.38	83.33	84	0.87 (0.74, 0.99)	0.09
Tp1 + Tp2		94.59	92.31	97.22	85.71	94	0.98 (0.94,1.0)	0.0003

The data were tumor contours based on post-contrast-enhanced MRI with morphological dilation. The numbers in parenthesis show the 95% confidence intervals

**Fig. 2:**
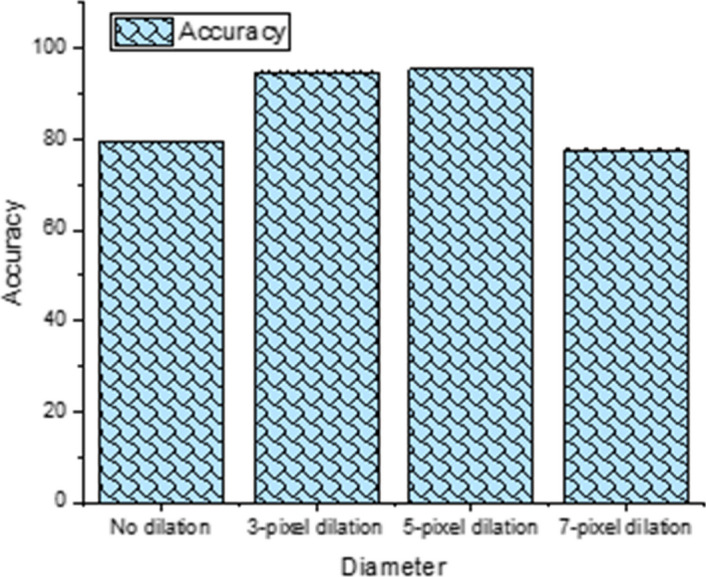
Accuracy for MRI texture of tp1 + tp2 data + molecular subtypes at different dilation voxel diameters

To evaluate the contribution of peri-tumor to prediction performance, we analyzed data without dilation, and with 3, 5, and 7-pixel dilation (Fig. 2). The data were those of tp1 + tp2 with inclusion of molecular subtypes. The performance of both 3- and 5-pixel dilation yielded better accuracy than that with no dilation and the 7-pixel dilation. The performance of 3- and 5-pixel dilation was also similar to each other. The performance of 7-pixel dilation was similar to that with no dilation.

**Fig. 3:**
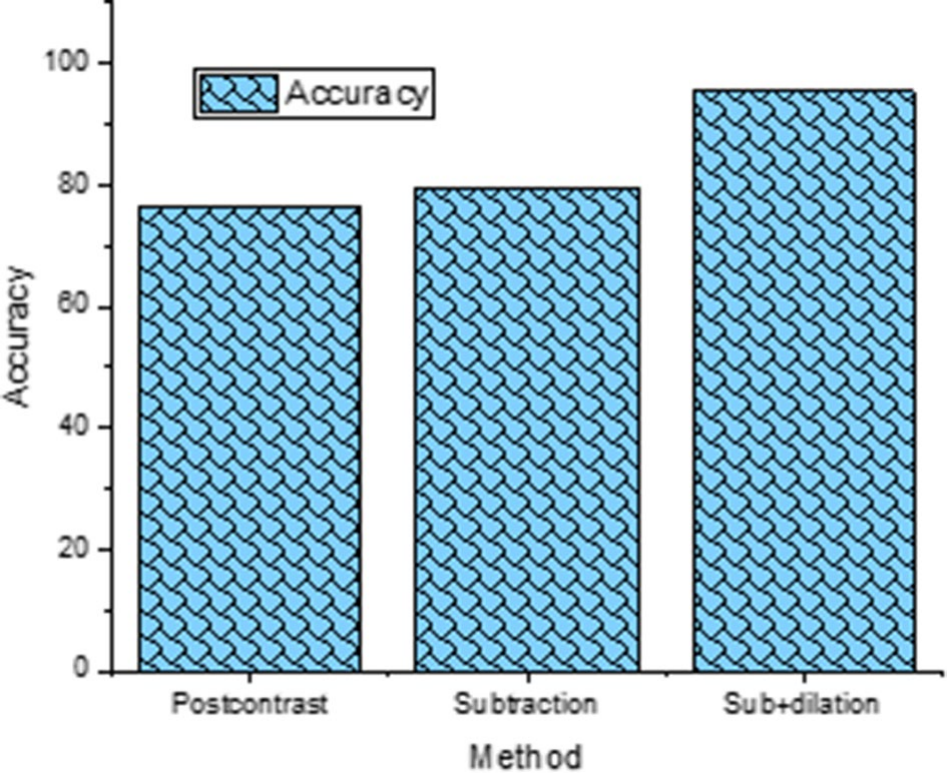
Accuracy for MRI texture analysis of tp1 + tp2 data + molecular subtypes for using image data of post-contrast MRI, subtraction of pre- and post-contrast MRI, and 5-pixel dilation of the subtracted images

We further compared the performance using image data of post-contrast MRI with that of subtraction for pre- and post-contrast MRI, and 5-pixel dilation of the subtracted images (Fig. 3). The results showed that the subtraction images and 5-pixel dilation of the tumor mask yielded the highest performance accuracy (94%). We also evaluated subtraction images with 3 and 7 pixels (data not shown); the accuracies were 93.8 and 78%, respectively.

Table 3 shows the performance comparison for post-contrast MRI, pre- and post-contrast subtraction images, and subtraction image with dilation. The AUC of the subtraction image with dilation was the highest, followed by subtraction image, and post-contrast MRI only. Similar conclusions were reached for other performance metrics. The *p*-values of the subtraction image with dilation was also the lowest.

**Table 3:** ROC metrics for predicting PCR using post-contrast image, subtraction image, and subtraction image with dilation at tp1 + tp2 using Ensemble RUSBoosted Tree classifier

Method	Sens.	Spec.	PPV	NPV	Accuracy	AUC	*P*-value
Post-contrast image	89.18	53.84	84.61	63.64	80	0.68 (0.48, 0.87)	0.212
Subtraction image	89.18	61.54	86.84	66.66	82	0.83 (0.70, 0.97)	0.128
Subtraction + 5 pixel dilation	94.59	92.31	97.22	85.71	94	0.98 (0.94, 1.0)	0.00029

The numbers in parenthesis show the 95% confidence intervals

Table 4 shows the results of PCR prediction performance of MRI texture + molecular subtypes using 5 machine-learning classifiers: Ensemble, KNN, SVM, Naïve Bayes, and Decision Tree Fine. The data were tumor contours without morphological dilation for combined tp1 + tp2. The Ensemble classifier yielded the highest prediction accuracy based on both accuracy and AUC.

**Table 4:** MRI texture analysis using combined tp1 + tp2 MRI data and molecular subtypes using different machine learning classifiers

Method	Sens.	Spec.	PPV	NPV	Accuracy	AUC	*P*-value
Rusboosted Tree	94.59	92.31	97.22	85.71	94	0.98 (0.94, 1.0)	0.00029
Decision Tree	1.00	0	74.00	NA	90	0.92 (0.81, 1.0)	0.00459
SVM coarse Gaussian	94.59	0	72.92	0	74	0.72 (0.55, 0.88)	0.5738
Kernel Naïve Bayes	70.27	69.23	86.66	45.00	70	0.70 (0.55, 0.85)	0.7925
KNN	94.59	0	72.92	0	70	0.60 (0.43, 0.76)	0.7924

The number in parenthesis showed the 95% confidence intervals. These data were tumor contour without morphological dilation

Table 5 shows the PCR prediction performance analysis on the tumor contour based on the post-contrast MRI with morphological dilation using Ensemble RUSBoosted Tree classifier based on single view and multiview methods without using SMOTE. The AUC of PCR prediction using molecular subtype was 0.82. The AUC of PCR prediction using MRI texture only at tp1, tp2 were 0.88, and 0.72, respectively. By contrast tp1 + tp2 yielded markedly better performance with an AUC of 0.96. Based on the Multiview technique the AUC of PCR prediction was 0.96 with slighter wider confidence interval with lower bound 0.91 and upper bound 1.00 and increased accuracy of 94.0.

**Table 5:** ROC metrics for predicting PCR based on molecular subtypes, MRI features at pre- and during NAC using Ensemble RUSBoosted Tree classifier based on single view and multiview without SMOTE

Time point	Features type	Sens.	Spec.	PPV	NPV	Accuracy	AUC
(View 1)	Molecular subtypes	86.48	76.92	91.42	66.66	84	0.82 (0.66, 0.97)
Tp1 (view 2)	MRI texture only	86.48	84.62	94.12	68.75	86	0.88 (0.77, 1.0)
Tp2 (view 3)		97.30	38.46	81.82	83.33	82	0.72 (0.53, 0.91)
Tp1 + Tp2 (view 4)		1.00	76.92	92.50	1.00	84	0.96 (0.92, 1.00)
Multiview	Molecular subtype and MRI texture	97.0	85.0	94.70	91.70	94.0	0.96 (0.91, 1.0)

The data were tumor contours based on post-contrast-enhanced MRI with morphological dilation. The numbers in parenthesis show the 95% confidence intervals

Table 6 shows the PCR prediction performance analysis on the tumor contour based on the post-contrast MRI with morphological dilation using Ensemble RUSBoosted Tree classifier based on single view and multiview methods with SMOTE. The AUC of PCR prediction using molecular subtype was 0.69. The AUC of PCR prediction using MRI texture only at tp1, tp2 were 0.86, and 0.76, respectively. By contrast tp1 + tp2 yielded performance with an AUC of 0.88. However, based on the Multiview technique using SMOTE, contrast tp1 + tp2 yielded markedly better performance with an **AUC of 0.98**.

**Table 6:** ROC metrics for predicting PCR based on molecular subtypes, MRI features at pre- and during NAC using Ensemble RUSBoosted Tree classifier based on single view multiview techniques with SMOTE

Time point	Features type	Sens.	Spec.	PPV	NPV	Accuracy	AUC
(View 1)	Molecular subtypes	65.0	69.0	85.70	40.90	66.0	0.69 (0.54, 0.90)
Tp1 (view 2)	MRI texture only	68.0	100	100	52.0	76.0	0.86 (0.77, 0.96)
Tp2 (view 3)		70.0	62.0	83.90	42.10	68.0	0.76 (0.58, 0.94)
Tp1 + Tp2 (View 4)		73.0	92.0	96.40	54.50	78.0	0.88 (0.78, 0.97)
Multiview	Molecular subtype and MRI texture	84.0	100	100	68.40	88.0	0.98 (0.94, 1.00)

The data were tumor contours based on post-contrast-enhanced MRI with morphological dilation. The numbers in parenthesis show the 95% confidence intervals

As different views contain information that describes a particular aspect of data, it is obvious that single-view data may contain incomplete knowledge, while multi-view data usually contains complementary, which results in a more accurate description of the data. Integrating the information contained in multiple views and by balancing the data with SMOTE helps to tune the class distribution that positively affect models in seeking good splits of the data during training. Hence, it improves the performance in case of multiview as compared to single view.

## Discussion

This study evaluated whether machine learning classification of texture features from breast MRI data obtained at different treatment time points in conjunction with molecular subtypes could accurately predict PCR associated with NAC in breast cancer. The major findings are: (i) molecular subtypes alone yield moderate prediction performance of eventual PCR; (ii) pre-, early and mid-treatment data alone also yield moderate performance; (iii) the combined pre- and early treatment data markedly improves prediction performance; (iv) the addition of molecular subtypes data improves performance slightly for individual time point data, and substantially improves performance for combined pre- and early treatment MRI; (v) the optimal morphological dilation was 3–5 pixels; (vi) post- and pre-contrast subtraction image with morphological dilation further improves performance, and (vii) among the machine-learning algorithms studied, RUSBoosted Tree machine-learning method yields the highest performance.

Molecular subtypes alone yielded moderate prediction accuracy of PCR, consistent with previous findings [4–6]. Pre-, early, and mid-treatment MRI data alone yielded moderate PCR prediction accuracy, consistent with a previous study that used tumor volumes at different time points to predict PCR [7] in which they found an AUC at pre- and post-treatment time points to be 0.7 and 0.73, respectively. Our study differed from the study by Hylton et al. [7] and most previous studies [12–17] in that we used machine-learning classification and we incorporated additional input parameters (such as molecular subtypes, data of different time points, and peri-tumoral features among others) into our prediction model.

The combined pre- and early treatment MRI data markedly improved prediction performance with an AUC of 0.98. A similar study by McGuire et al. also showed that MRI data from two time points predicted PCR moderately well (AUC = 0.777) [19]. Our study differed from McGuire’s in that our approach included molecular subtypes. The addition of molecular subtypes data only moderately improved performance of individual time points, but substantially improved performance of the combined tp1 + tp2 data. The *p*-value was markedly smaller for the combination time points when molecular subtypes were incorporated into the model. This finding further supports the notion that longitudinal changes in texture features helps to improve the PCR prediction performance.

Peritumoral microenvironment could affect chemoresistance [18]. It is not surprising that peri-tumoral image features are relevant in predicting treatment response [13]. We evaluated texture features with graded morphological dilation to assess the contribution of peri-tumoral areas to prediction performance. We found that the performance had an inverted U-shape curve with 3–5-pixel dilation yielding optimal prediction performance. This is not unexpected because too little dilation is not expected to be helpful and too much dilation would include normal tissue which is not helpful either. Similarly, the performance of the subtraction image with dilation outperformed both post- and subtraction images. This is not unexpected because edge detectors work by dilating an image and then subtracting it away from the original to highlight those new pixels at the edges of the object of interest.

We compared the prediction performance of 5 machine learning classifiers. The Ensemble classifier yielded the highest prediction accuracy and AUC, followed by the Decision Tree classifier. Mani et al. [20] investigated PCR in 20 patients after a single cycle of NAC using the following classifiers: Gaussian Naïve Bayes, logistic regression, and Bayesian logistic regression two Decision Tree-based classifiers (CART36 and Random Forest), one kernel-based classifier (SVM), and one rule learner (Ripper). They showed that imaging and clinical parameters boosted the performance of Bayesian logistic regression. Qu et al. [12] predicted PCR to NAC in breast cancer with two combined time points, using a multipath deep convolutional neural network and obtained a similar AUC as our current study. However, they did not include molecular subtypes. Tahmassebi et al. applied eight machine learning classifiers using a single time point and attained a high AUC using XGboost disease-specific survival (DSS) as the standard of reference [21]. Their results suggest that the choice of classifier is important in determining accuracy; XGboost classifier is also among the top performers.

AI-classification of texture features from MRI of breast tumor at multiple treatment time points accurately predicts eventual PCR. Longitudinal changes in texture features and peri-tumoral features further improve PCR prediction performance. Accurate assessment of treatment efficacy early on could minimize unnecessary toxic chemotherapy and enable mid-treatment modification for patients to achieve better clinical outcomes. Because PCR is associated with recurrent free survival, this approach also has the potential to improve quality of life. Novelty is that the model used multiple time point MRI data and non-imaging data to improve PCR prediction accuracy. Analysis of peri-tumor by graded dilation was also evaluated. Multiple machine learning models were evaluated.

This study had a several limitations. This is a retrospective multicenter study. The sample size is relatively small. These findings need to be replicated in a prospective study with a larger sample size. This study used supervised machine learning of texture feature. Future work could use deep-learning artificial intelligence methods.

## Conclusion

Machine learning classification of texture features from MRI of breast tumor at combined time points of treatment can accurately predict pathologic complete response. Specifically, inclusion of molecular subtypes, longitudinal changes in texture features and peri-tumoral features improve the PCR prediction performance. This accurate assessment of treatment efficacy early on could minimize unnecessary toxic chemotherapy and enable mid-treatment modification to achieve better clinical outcomes.

## Methods

### Patient cohort

Patients from the I-SPY-1 TRIAL (2002–2006) were used [7, 22, 23]. All patients had locally advanced stage 2 or 3 unilateral breast cancer with breast tumors ≥ 3 cm in size and underwent anthracycline–cyclophosphamide and taxane treatment. MRI was acquired pre-NAC (pre-treatment, time point 1 [tp1]), ~ 2 weeks after the first cycle of anthracycline–cyclophosphamide (early treatment, tp2), after all anthracycline–cyclophosphamide was administered but before taxane (mid-treatment, tp3), and post-NAC and before surgery (post-treatment, tp4). In addition to imaging data, molecular subtypes were also included in the prediction model, and they included HR+/HER2−, HER2+, with triple negative, HR+/−, PgR+/−, ER+/−, and level of Ki67 (see Table 1 for definition of abbreviations). Full patient characteristics and demographic data are shown in Table 1. There is a total *n* = 121 patients in the available dataset, however a clinical and outcome excel file referring sheet TCIA outcome subset contains PCR for *n* = 166 subject only. The remaining subjects had missing data and were not applicable for PCR prediction. Patients available at (https://wiki.cancerimagingarchive.net/display/Public/ISPY1). The ground truth was PCR status, defined as complete absence of invasive cancer in the breast and axillary lymph nodes after NAC. The sample size was 166 patients divided into two classes based on PCR status. 124 achieved full PCR and 42 had residual cancer after NAC and did not achieve full PCR. The results were computed with and without SMOTE to handle the imbalance of the data. Moreover, the *k*-fold cross-validation technique was employed to handle the overfitting.

### MR imaging protocol

The MR imaging protocol as detailed in [24, 25] was used in our study according to the below information. MR imaging was performed by using a 1.5-T field strength MR imaging system and a dedicated four- or eight-channel breast radiofrequency coil. Patients were placed on the MR imaging table in the prone position with an intravenous catheter inserted within the antecubital vein or hand. The image acquisition protocol included a localization sequence and a T2-weighted sequence followed by a contrast-enhanced T1-weighted series. All imaging was performed unilaterally over the symptomatic breast in the sagittal orientation. The contrast-enhanced series consisted of a high-resolution (1 mm in-plane spatial resolution), three-dimensional, fat-suppressed, T1-weighted gradient-echo sequence (20 ms repetition time, 4.5 ms echo time, 45° flip angle, 16 × 16 cm to 20 × 20 cm field of view, 256 × 256 matrix, 60–64 slices, 1.5–2.5 mm slice thickness). The imaging time length for the T1-weighted sequence was between 4.5 and 5 min. The sequence was performed once before the injection of a contrast agent and repeated two to four times after injection of agent.

### Preparation of images

The tumor on the first post-contrast image (~ 2 min post-contrast MRI) was manually segmented with ITK-SNAP, into tumor masks by a trainee who was supervised and reviewed by an experienced breast radiologist (20+ year of experience). Images with poor visualization of breast tissue were excluded (usually due to poor contrast visualization or faulty fat suppression). In addition, we also calculated subtraction images from the pre- and first post-contrast MRI. A peri-tumor mask was obtained using the standard morphological dilation operation with the spherical structural element containing 3, 5, and 7 voxels as its diameter using Matlab2019b. Dilated images were obtained for post-contrast as well as subtraction images.

### Overview of PCR prediction analysis

This study specifically aimed to predict PCR based on several criteria detailed below and quantify their contribution to the model. Specifically, the criteria were multiple texture features, incorporating molecular subtype, combining images from different time points, performing morphological dilation, computing performance based on single and multiview, and handling the imbalanced data with synthetic minority oversampling (SMOTE). First**,** we performed PCR prediction using: (i) all molecular subtypes data alone; (ii) MRI tumor texture features only at pre-treatment time point using segmented tumor of post-contrast MRI; (iii) MRI tumor texture features + molecular subtypes; (iv) texture analysis using pre-treatment, different time points during NAC, and their combinations by concatenating the time points; (v) texture analysis of peri-tumor mask with graded morphological dilation; (vi) post- and pre-contrast subtraction MRI, and (vii) morphological dilation of the subtraction MRI. We also compared the prediction performance of 5 machine learning classifiers: Ensemble, *K*-nearest neighbor, support vector machine, Naïve Bayes, and Decision Tree. We optimized the machine learning algorithms to find the best combination for our type of data. We also computed the performance based on single view and multiple view with and without SMOTE technique.

#### Multiview representation

In real-world problems, the machine learning applications are used in which we can use multiple ways to represent the features using clustering, classification and feature learning as detailed in [26–32]. In this study, we used multiview classification with different feature learning and time points, i.e., imaging with different feature types, non-imaging, multiple time points, etc., of an example. For example, a webpage contains words in the page, which can also contain hyperlinks which refer to it from other pages. Likewise, internet images can also be reflected by the visual features within it, and text which surround it. In multiple view representation, we simply concatenate different features into single one. To represent the multiview approach, a commonly known method such as multi-view learning (MVL) is of great interest of the recent years [31, 33–37]. There are many approaches discussed in [29, 33, 38–40] which reflect the multiple view learning approach is better than the naïve approach which use one view or concatenating all view. Xu et al. [30] discussed the MLV methods with two significant principles: consensus and complementarity. (1) Consensus principles maximize the agreement among multiple view. A co-regularization method [41] is used to minimizes the distance between the predictive function of two views as well as the loss within each view. There are various methods from the family of co-regularization style methods [39, 42] considered after the consensus principle. (2) The complementarity principle assumes each view of data contains some information does not present in the other view. Thus, accurately and comprehensively utilizing information from multiple view is expected to produce better models. The probabilistic latent semantic analysis [43] using multiview approach model jointly the co-occurrences of features and documents from different views and utilized two conditional probability to capture the specified structures inside each view to model the complimentary information. The maximum entropy discrimination (MED) [44] method is used to integrate the two principles into a single framework for Multiview approach. MED is widely used in many applications such as feature selection [45], classification [28], structured prediction [42, 44, 46], multi-task learning [47]. Moreover, multi-view maximum entropy discrimination (MVMED) [48] extends MED to MVL. The MVMED method makes full use of all Multiview information of the data by considering the two common principles such as complementarity and consensus.

**Fig. 4:**
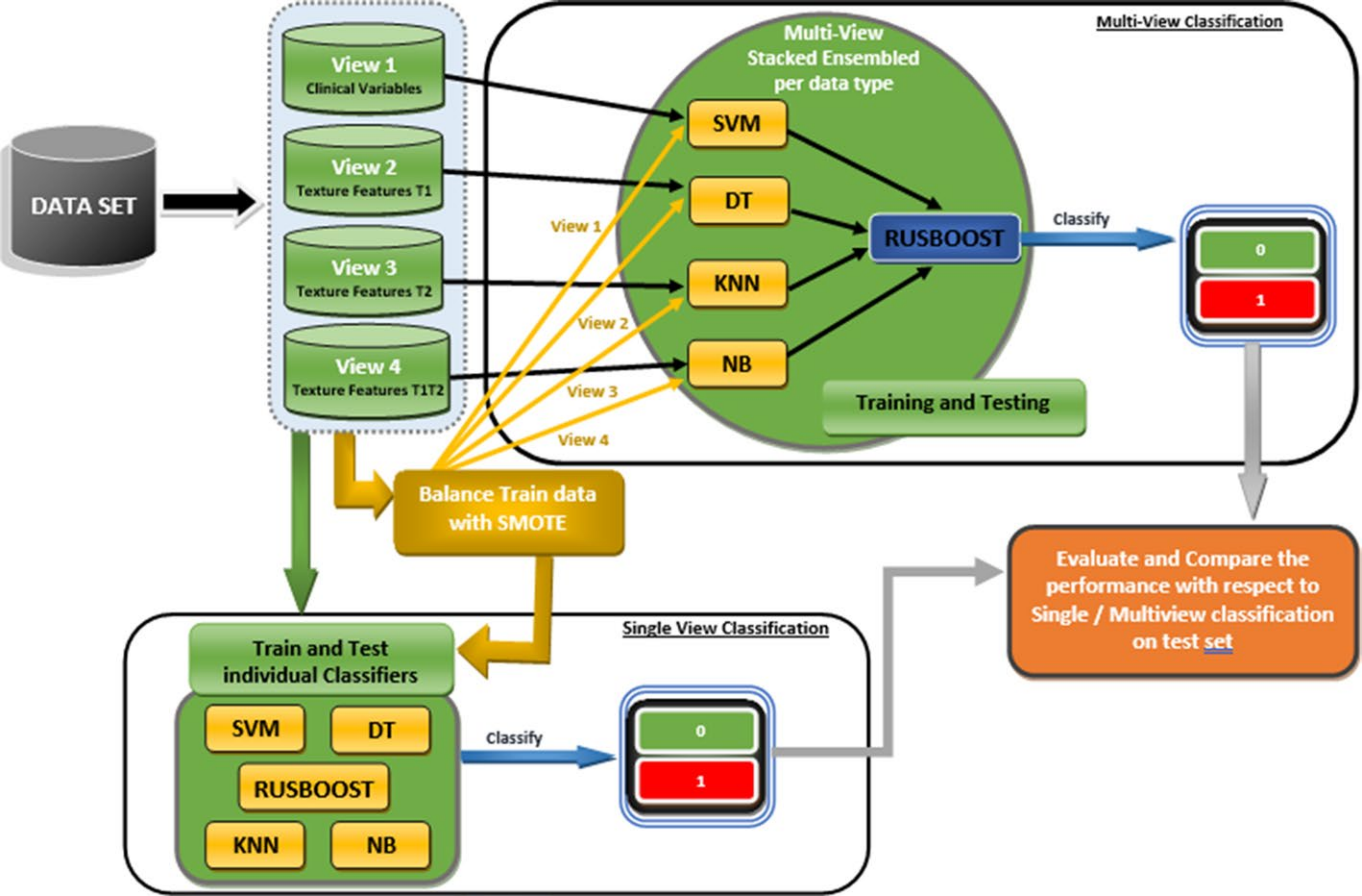
Schematic diagram to show the flow of our model for prediction of PCR with single and multiview classification techniques and with and without SMOTE method

Figure 4 reflects the flow of work. In this research work, we first used multi-view stacked Ensemble approach by categorizing the data into different views where view 1 contain the Molecular subtype variables, view 2 contains the textural feature of MRI at first time point (T1), view 3 have textural feature of MRI at 2nd time point (T2), whereas view 4 contains textural features of T1 and T2 after concatenation. Then, we split the each view into train and test data. The training data of each view train were used to train classifiers as per data view (i.e., SVM on View 1, DT on View 2, KNN on View 3 and NB on View 4) and prediction probabilities of each classifier are used together to create a low dimensional dataset which is then used to train our final classifier (i.e., RUSBOOST). In contrast, we also applied SMOTE on the training data of each view to balance the dataset. Then we applied the similar process as discussed above for normal train data of each view.

For single view, we also train the classifiers on each view individually with and without applying SMOTE on train data of all views. Finally, we evaluated the performance on all classifiers on test data of each view using different performance metrics with respect to respect to single/multi-view classification.

### Texture features

The texture features are estimated from the Grey-level Co-occurrence Matrix (GLCM) [49–51] covering the pixel (image) spatial correlation. Each GLCM input image $\left( {{\mathbf{u}},{\mathbf{v}}} \right){\text{th}}$ defines how often pixels with intensity value ${\mathbf{u}}$ co-occur in a defined connection with pixels with intensity value ${\mathbf{v}}$. We extracted second-order features consisting of contrast, correlation, mean, entropy, energy, variance, inverse different moment, standard deviation, smoothness, root mean square, skewness, kurtosis, and homogeneity previously used in [52–58]*.*

### Classification

We applied and compared 5 supervised machine learning classification algorithms: Ensemble, *K*-nearest neighbor (KNN), SVM coarse Gaussian, Kernel Naïve Bayes, and Decision Tree Fine. The ensemble includes the RUSBoosted method (random undersampling boosting) which is a hybrid data sampling/boosting algorithm which can eliminate data distribution imbalance between the classes and improve the classification performance of the weak classifiers [59, 60]. KNN is the most widely used algorithm in the field of machine learning, pattern recognition, and in many other areas [61]. A model or classifier is not immediately built, but all training data samples were saved and waited until new observations are needed to be classified. This characteristic of the lazy learning algorithm makes it better than eager learning which constructs a classifier before new observations need to be classified. SVM Coarse Gaussian (SVMCG) is a nonlinear SVM learning technique used for optimization tasks and prediction of new data sets from a few given samples [62, 63]. The coarse Gaussian kernel is a fitness function that makes the computation process easier and faster [64]. This algorithm works with fast binary and hard medium, slow and large multiclass, and kernel scale set [65]. The Kernel Naïve Bayes (KNB) [66] algorithm is based on the Bayesian theorem [67] and is suitable for higher dimensionality problems. This algorithm is also suitable for several independent variables whether they are categorical or continuous. Moreover, this algorithm can be a better choice for average higher classification performance and minimal computational time in constructing the model. In Decision Tree algorithms, the score generated by each Decision Tree for each observation and class is the probability of this observation originating from this class computed as the fraction of observations of this class in the tree leaf. All classification algorithms were performed using Matlab (R2019b, MathWorks classification App) with typical default parameters used for each of the classifiers. Following algorithms were used for classification:

#### Support vector machine

Support vector machine (SVM) is the most important technique of supervised learning methods, which is also used for classification purposes. For solving the problems related to pattern recognition [68], medical analysis area [69, 70], and machine learning [71] recently SVM are used. Furthermore, SVMs are also used in many other fields such as detection and recognition, recognizing of text, image retrial based on contents, biometric systems and speech recognition, etc. To build a single hyperplane or set of hyperplanes in infinite space or high dimension, SVM is used. For classifying a good classification this hyperplane may also be used. By implementing this, a hyperplane which has the greatest distance to nearby training point of any class is achieved. Usually, lower generalization fault of the classifier is achieved by larger margin.

Support vector machine tries to find a hyperplane that gives the training example with greatest minimum distance. In Support vector machine theory, this is also termed as margin. For maximized hyperplane the best margin is attained. There is additional significant characteristic for SVM that provides the better generalization results. Support vector machine mainly has a two-type classifier which converted data into a hyperplane dependent on data that is nonlinear or dimensionally higher.

#### Kernel trick

The data which is not linearly separable, Müller et al. [72] recommended kernel trick to handle this type of data. To cope up with this type of problem, the nonlinear mapping function from the input space is transformed into higher dimensional feature space. Thus, in the input space, the dot product between two vectors is expressed by the dot product with some kernel functions in the feature space. The SVM coarse Gaussian kernel was trained data that comprised input variable (*x*) as predictors and output variable (*y*) as responses and cross-validation is performed.

Below equations display the mathematical representation of the coarse Gaussian kernel and the kernel scale set, where *P* is the number of predictors:

$$ {\text{Gaussian}}\;{\text{Kernel:}}\;K\left( {x,x_{i} } \right) = e^{{ - y~\left| {x - x_{i} } \right|^{2} }} , $$

$$ {\text{Kernel}}\;{\text{Scale}}\;{\text{set:}}\;\left( P \right)*4. $$

#### Decision Tree (DT)

The DT classifier checks the dataset similarity that is given and classifies it into different separate classes. Decision Trees are used for making classifiers of data depending on the choice of a feature which fixes and maximizes the data division. These attributes are separated into different branches until the end criteria are met.

The Decision Tree classifier is based on supervised learning technique, which used a recursive approach by dividing dataset in order to reach at a similar classification. Most of the classification problems with large data sets are complex and contain errors, the Decision Tree algorithm is most appropriate in these situations. The Decision Tree works by taking the objects as an input and give output as yes/no decision. Decision Trees use sample selection [73] and also exhibit Boolean functions [74]. The Decision Trees are also quick and effective methods used for large classification data set entries and provide best decision support proficiencies. There are many applications of using DTs such as medical problems, economic and other scientific situations, etc. [75]*.*

#### *K*-nearest neighbor (KNN)

In the field of pattern recognition, machine learning and other different fields, *K*-nearest neighbor is regularly utilized algorithm. KNN is non-parametric method used for both classification and regression problems. In both of these cases, the given input consists of *k*-closest training samples in the feature space. The output is dependent that whether we use KNN for regression or classification. For KNN classification method, the output is a class membership. Any object can be classified based on the majority voting of its neighboring data points with the object being assigned to the class that is common among its *k*-nearest neighbors (where *k* is a positive integer, typically small). If *k* = 1, then the objects will be classified and assigned to the nearest class of that single neighbors.

The *k*-nearest neighbors (*k*-NN) algorithm is a non-parametric technique that is used for regression and classification purposes. In both mentioned cases, the given input comprises the *k*-closest training samples in the feature space. The received output is dependent on whether we are using *k*-NN for regression or classification purpose. In *k*-NN classification method, the output is a class membership. On the basis of majority voting of its neighboring data points any object is classified, with the object being assigned to the class that is common among its *k*-nearest neighbors (*k* is a positive integer, typically small). If we suppose that *k* = 1, then the object will be simply classified and assigned to the nearest class of that single neighbor. We used the default parameters during training/testing of data using KNN algorithm. KNN was used for classification complications in [76]. KNN is also termed lazy learning algorithm. A classifier is not promptly constructing however all preparation information tests are spared and held up until the point that new perceptions should be classified. Due to these characteristics of lazy learning algorithm it marks better than excited learning, because it builds a classifier previously new interpretations need to be classified. It is explored by [77] that KNN is also more important when it is required to change the dynamic data and more rapidly simplified. Different distance matrices are employed for KNN.

#### Kernel Naïve Bayes (KNB)

The Naïve Bayes classifier is successfully been used in many of the classification problems successfully, however most recently, the Al-khurayji and Sameh used latest kernel function [78] for classification of Arabic text by yielding the most effective results. Likewise, Bermejo, Gámez, and Puerta employed [79] for feature selection in an incremental wrapper function.

The Naive Bayes classifier is a simple and efficient stochastic classification method and is based on Bayesian theory based on supervised classification technique. For each class value, it estimates that a given instance belongs to that class [80, 81]. A feature item of a class is independent of other feature values called class conditional independence. In machine learning, from the family of probabilistic classifiers, Naïve Bayes [82] classifier was used which is based on the Bayes’ theorem having strong independence assumptions between the features. NB is most popular in classification tasks [83]. This algorithm is most popular since 1950. Due to the good behavior [84], NB is extensively used in recent developments [79, 85–88] which try to improve NB performance.

#### RUSBoosted Tree

The ensemble classifiers comprise a set of individually trained classifiers whose predictions are then combined when classifying the novel instances using different approaches [89]. These new learning algorithms by constructing set of algorithms classify new data based on the new data points by taking weight of their prediction. Based on these capabilities, these algorithms have successfully been used to enhance the prediction power in variety of applications such as predicting signal peptide for predicting protein subcellular location [90], predicting subcellular location and enzyme subfamily prediction [91]. The ensemble classifiers in many applications gives relatively enhanced performance than the individual classifier. The researchers [92] reported that individual classifiers during classification can produce different errors, however these errors can be minimized by combining classifiers because the error produced by one classifier can be compensated by the other classifier.

Mata boosting technique AdaBoost used for weak learners to improve classification performance by creating ensemble hypotheses iteratively, these weak hypotheses are combined to the unlabelled example. Error is also included with their weights so misclassified have increased weights and correctly classified have decreased weights. The authors in [93] enhance AdaBoost which increases classification accuracy of imbalance data by improving imbalanced class distribution. RUS is robust than AdaBoost as it reduces the class distribution imbalance problem of the training set [59, 94]. RUS is the most common and robust Data sampling method due to its simplicity, it intelligently performed under sampling and oversampling until the desired result is archived. Verma and Pal also employed different ensemble methods to classify skin diseases; the results reveal that ensemble methods yielded more accurate and effective skin disease predictions.

#### Synthetic minority oversampling (SMOTE)

The SMOTE is used to handle the imbalanced data. There exist numerous differences of distribution for the classification of datasets among the quantities of minority class and majority class, which is commonly known as imbalanced dataset. It is being considered as a challenging problem to learn from imbalanced datasets in supervised learning because a standard classification algorithm is designed to distribute the dataset in balanced proportion. For this purpose, oversampling is one of the best-known methods. Oversampling creates artificial data to get a balanced dataset distribution. Synthetic minority oversampling (SMOTE) is a kind of oversampling method that is most widely used to balance the imbalanced data in machine learning. SMOTE technique arbitrarily creates new instances of minority class from the nearest neighbors of the minority class. Furthermore, these instances are used to view the various features of original dataset and will be considered as original instances of the minority class [95, 96].

Several studies have shown significant results for the implementation of oversampling method. The combination of SMOTE with different classification algorithms has been implemented and have shown the improved performance of prediction systems, such as credit scoring, bankruptcy prediction, network intrusion detection and medical diagnosis. Region adaptive synthetic minority oversampling technique (RA-SMOTE) is proposed by Yan et al. and implemented in the detection of intrusion to recognize the attack in the network [97]. Sun et al. proposed another hybrid model by using SMOTE for unbalanced dataset to be utilized as a helping tool to evaluate the enterprise credit for bank [98]. Later, this system was applied to the financial data of 552 companies and outperformed than other earlier used traditional models. Le et al. employed several oversampling techniques to manage the unbalanced data problems related to financial datasets [99, 100].

In medical field, the combination of different classification algorithms with SMOTE has been widely used in disease classification and medical diagnosis. Wang et al. presented a hybrid algorithm by using well-known classifier, particle swarm optimization (PSO) and SMOTE to enhance the prediction of breast cancer from a huge imbalanced dataset [101].

SMOTE is a method for creating synthetic observations (not oversampling the observation by with-replacement sampling method) based on the minority observations that exist in the data set. The synthetic samples of minority class are over-sampled by taking each minority class sample and introducing synthetic examples along the line segments which join all or any of the k minority class nearest neighbors. So, it randomly picks up the number of samples that are needed, from the *k-*nearest neighbors. This approach minimizes the classifiers overfitting problem by broadening the minority class decision region.

SMOTE technique boosts the minority class set $S_{{\min }}$ by producing counterfeit samples based on the feature space similarities between existing minority samples. The SMOTE technique can be defined as:

For each sample $\user2{x}_{i}$ in $\user2{S}_{{\min }}$, let $\user2{S}_{\user2{i}}^{\user2{K}} ~$ be the set of the *K*-nearest neighbors of $\user2{x}_{\user2{i}}$ in $\user2{S}_{{\min }}$ according to the Euclidian distance metric. To produce a new sample, an element in $\user2{S}_{\user2{i}}^{\user2{K}}$, denoted as $\widehat{{\user2{x}_{\user2{i}} }}$, is selected and then multiplied by the feature vector difference between $\widehat{{\user2{x}_{\user2{i}} }}$ and $\user2{x}_{i}$ and by any randomly selected number between [0, 1]. Finally, the obtained vector is added to $\user2{x}_{i} :$

$$ \mathbf{x}_{{{\text{new}}}}  = \mathbf{x}_{\mathbf{i}}  + \left( {\widehat{{\mathbf{x}_{\mathbf{i}} }} - ~\mathbf{x}_{\mathbf{i}} } \right) \cdot \mathbf{\delta }, $$

where $\delta  \in \left[ {0,1} \right]$ is a random number.

These produced samples help to break the ties introduced by ROS and augment the original dataset in a manner that, in short, significantly enhances the learning process [102, 103].

### Performance evaluation measures

The performance was evaluated with the following parameters.

#### Sensitivity

The sensitivity measure also known as **TPR** or recall is used to test the proportion of people who test positive for the disease among those who have the disease. Mathematically, it is expressed as:

$$ {\text{Sensitivity}} = \frac{{\sum {\text{True}}\;{\text{positive}}}}{{\sum {\text{Condition}}\;{\text{positive}}}}, $$

$$ {\text{Sensitivity}} = \frac{{{\text{TP}}}}{{{\text{TP}} + {\text{FN}}}}, $$

i.e., the probability of positive test given that patient has disease.

#### Specificity

The TNR measure also known as **Specificity** is the proportion of negatives that are correctly identified. Mathematically, it is expressed as:

$$ {\text{Specificity}} = \frac{{\sum {\text{True}}\;{\text{negative}}}}{{\sum {\text{Condition}}\;{\text{negative}}}},~ $$

$$ {\text{Specificity}} = \frac{{{\text{TN}}}}{{{\text{TN}} + {\text{FP}}}}, $$

i.e., probability of a negative test given that patient is well.

#### Positive predictive value (PPV)

PPV is mathematically expressed as:

$$ {\text{PPV}} = \frac{{\sum {\text{True}}\;{\text{positive}}}}{{\sum {\text{Predicted}}\;{\text{condition}}\;{\text{positive}}}},~ $$

$$ {\text{PPV}} = \frac{{{\text{TP}}}}{{{\text{TP}} + {\text{FP}}}}, $$

where TP denotes that the test makes a positive prediction and subject has a positive result under gold standard while FP is the event that test make a positive perdition and subject make a negative result.

#### Negative predictive value (NPV)

NPV can be computed as:

$$ {\text{NPV}} = \frac{{\sum {\text{True}}\;{\text{negative}}}}{{\sum {\text{Predicted}}\;{\text{condition}}\;{\text{negative}}}}, $$

$$ {\text{NPV}} = \frac{{{\text{TN}}}}{{{\text{TN}} + {\text{FN}}}}, $$

where TN indicates that test make negative prediction and subject has also negative result, while FN indicate that test make negative prediction and subject has positive result.

#### Accuracy

The total accuracy is computed as:

$$ {\text{Accuracy}} = \frac{{{\text{TP}} + {\text{TN}}}}{{{\text{TP}} + {\text{FP}} + {\text{FN}} + {\text{TN}}}}.~ $$

#### Receiver operating curve (ROC)

The ROC is plotted against the true positive rate (TPR), i.e., sensitivity and false positive rate (FPR), i.e., specificity values of PCR and non-PCR subjects. The mean features values for PCR subjects are classified as 1 and non-PCR subjects are classified as 0. This vector is then passed to the ROC function, which plots each sample values against specificity and sensitivity values. ROC is a standard way to classify the performance and visualize the behavior of a diagnostic system [104]. The TPR is plotted against *y*-axis and FPR is plotted against *x*-axis. The area under the curve (AUC) shows the portion of a square unit. Its value lies between 0 and 1. AUC > 0.5 shows the separation. The higher AUC shows the better diagnostic system. Correct positive cases divided by the total number of positive cases are represented by TPR, while negative cases predicted as positive divided by the total number of negative cases are represented by FPR.

### Training/testing data formulation

The Jack-knife fivefold cross-validation technique with below steps as detailed in [105–107] was applied for the training and testing of data formulation and parameter optimization. It is one of the most well-known, commonly practiced, and successfully used methods for validating the accuracy of a classifier using a fivefold cross-validation. The data are divided into fivefolds in training, the fourfolds participate, and classes of the samples for remaining folds are classified based on the training performed on fourfolds. For the trained models, the test samples in the test fold are purely unseen. The entire process is repeated 5 times and each class sample is classified accordingly. Finally, the unseen samples classified labels that are to be used for determining the classification accuracy. This process is repeated for each combination of each system’s parameters and the classification performance indices were computed.

#### Handling the overfitting problem

**Fig. 5:**
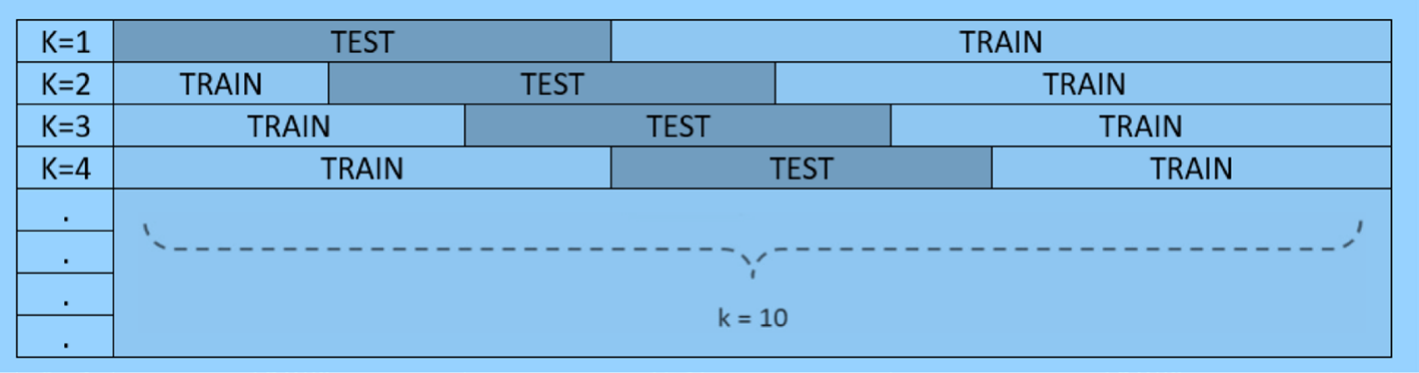
*K*-fold cross-validation procedure to avoid the model for overfitting

*K*-fold cross-validation as shown in Fig. 5 is an effective preventative measure against overfitting. Thus, to tune the model, the dataset is split into multiple train-test bins. Using *k*-fold CV, the dataset is divided into *k*-folds. For model training, *k* − 1 folds are involved, and rest of the folds are used for model testing. Moreover, *k*-fold method is helpful for fine-tuning the hyperparameters with the given original training dataset in order to determine that how the outcome of ML model could be generalized. The *k*-fold cross-validation procedure is reflected in Fig. 5 below. In this research work, we kept the value of *k* = 10, i.e., tenfold cross-validation is used to avoid the overfitting problem, where the final performance of the models trained on the tenfold CV is tested using testing samples set consisting of non-augmented values (without SMOTE) to evaluate performance on actual data (non-augmented data).

### Statistical analysis and performance measures

Analyses examining differences in outcomes across different time points used unpaired 2-tailed *t*-tests with unequal variance [108, 109]. Receiver operating characteristic (ROC) curve analysis [110, 111] was performed with PCR as ground truth. AUCs with lower and upper bounds and accuracy were tabulated. Matlab (R2019b, MathWorks, Natick, MA) was used for statistical analyses. The performance was evaluated in terms of sensitivity, specificity, positive predictive value (PPV), negative predictive value, accuracy, area under the receiver operating characteristic (AUC) curve as detailed in [105–107].

## Data Availability

These data are already available via https://wiki.cancerimagingarchive.net/display/Public/ISPY1.

## Abbreviations

PCR: Pathological complete response
NAC: Neoadjuvant chemotherapy
MRI: Magnetic resonance imaging
ER: Estrogen receptor
PgR: Progesterone receptor
HR: Hormone receptor
HER2: Human epidermal growth factor receptor 2
Tp1: Time point 1
Tp2: Time point 2
RUSBoosted: Random undersampling boosting
PPV: Positive predictive value
NPV: Negative predictive value
AUC: Area under curve
CV: Cross-validation
ROC: Receiver operating characteristic
SVM: Support vector machine
*k*-NN: *K*-Nearest neighbors
KNB: Kernel Naïve Bayes
DTs: Decision Trees
XGBoost: EXtreme Gradient Boosting
CART: Classification and Regression Tree
RF: Random Forest
DSS: Disease-specific survival
